# Laforin targets malin to glycogen in Lafora progressive myoclonus epilepsy

**DOI:** 10.1242/dmm.049802

**Published:** 2023-01-06

**Authors:** Sharmistha Mitra, Baozhi Chen, Peixiang Wang, Erin E. Chown, Mathew Dear, Dikran R. Guisso, Ummay Mariam, Jun Wu, Emrah Gumusgoz, Berge A. Minassian

**Affiliations:** ^1^ Division of Neurology, Department of Pediatrics, University of Texas Southwestern Medical Center, Dallas, TX 75390, USA; ^2^ Program in Genetics and Genome Biology, The Hospital for Sick Children Research Institute, Toronto, ON M5G 0A4, Canada; ^3^ Department of Pathology, University of Texas Southwestern Medical Center, Dallas, TX 75390, USA

**Keywords:** Polyglucosan, Lafora body, Myoclonus

## Abstract

Glycogen is the largest cytosolic macromolecule and is kept in solution through a regular system of short branches allowing hydration. This structure was thought to solely require balanced glycogen synthase and branching enzyme activities. Deposition of overlong branched glycogen in the fatal epilepsy Lafora disease (LD) indicated involvement of the LD gene products laforin and the E3 ubiquitin ligase malin in regulating glycogen structure. Laforin binds glycogen, and LD-causing mutations disrupt this binding, laforin–malin interactions and malin's ligase activity, all indicating a critical role for malin. Neither malin's endogenous function nor location had previously been studied due to lack of suitable antibodies. Here, we generated a mouse in which the native malin gene is tagged with the FLAG sequence. We show that the tagged gene expresses physiologically, malin localizes to glycogen, laforin and malin indeed interact, at glycogen, and malin's presence at glycogen depends on laforin. These results, and mice, open the way to understanding unknown mechanisms of glycogen synthesis critical to LD and potentially other much more common diseases due to incompletely understood defects in glycogen metabolism.

## Teaser

**Summary:** A new FLAG-malin mouse shows that E3 ubiquitin ligase malin associates with glycogen via laforin, uncovering molecular bases of unknown glycogen metabolism critical to Lafora disease and potentially common diseases.

## INTRODUCTION

Lafora disease [LD; Online Mendelian Inheritance in Man (OMIM) #254780] is a neurodegenerative epilepsy that afflicts previously healthy teenagers. Underlying it is a continuous formation of glycogen molecules with overlong and overphosphorylated branches called polyglucosans, which precipitate and gradually aggregate into ever larger and more numerous inclusions called Lafora bodies (LBs). These drive escalating neuroinflammation and neurodegeneration, leading to progressive intractable epilepsy and dementia, and death within 10 years of onset (Mitra et al., 2022).

Laforin (*EPM2A*) binds glycogen through a canonical carbohydrate-binding domain (CBM20) and scaffolds Mst1 (Stk4) and Mst2 (Stk3) to glycogen (Ganesh et al., 2004; Kuchtová et al., 2018; Liu et al., 2021; Wang et al., 2002). Oversequestration of these tumor suppressor proteins at glycogen lifts their inhibition of the Yap oncoprotein and drives several cancers (Liu et al., 2021). Laforin dephosphorylates glycogen through a dual-specificity phosphatase domain (Ganesh et al., 2000; Tagliabracci et al., 2007; Worby et al., 2006). Finally, laforin appears to bind the E3 ubiquitin ligase malin (*NHLRC1*) (Gentry et al., 2005; Lohi et al., 2005; Sánchez-Martín et al., 2015; Solaz-Fuster et al., 2008; Vilchez et al., 2007; Worby et al., 2008), although this has not been confirmed in the native state. Mutations in the laforin or malin genes that disrupt laforin's binding to glycogen, the putative laforin–malin interaction or malin's ubiquitin ligase activity cause LD (Brewer et al., 2021; Chan et al., 2003; Fernandez-Sanchez et al., 2003; Ganesh et al., 2004; Gentry et al., 2005; Minassian et al., 1998; Wang et al., 2002). Introduction of phosphatase-inactive laforin into laforin-knockout LD mice does not correct glycogen overphosphorylation, but does correct glycogen chain lengths and prevents polyglucosan and LB formation (Gayarre et al., 2014). This implied that phosphatase activity of laforin is dispensable to rescue the LD phenotype in laforin-deficient mice. Introduction of phosphatase-inactive laforin into malin-knockout LD mice does not correct glycogen overphosphorylation or chain overelongation, and does not prevent polyglucosan and LB formation (Gayarre et al., 2014; Nitschke et al., 2017). Taken together, these results suggest that laforin and malin work together to regulate glycogen structure through an unknown mechanism in which malin's ubiquitin ligase activity is critical. Whether this mechanism operates at glycogen itself or elsewhere is also unknown. Further inroads into this previously unrecognized facet of glycogen metabolism could not be made primarily because all attempts to generate an adequate antibody to study native malin have so far not succeeded. In the present work, we generated and characterized a mouse in which the malin gene is tagged with the FLAG epitope DNA sequence. Among other results, we show that the FLAG-malin gene expresses physiologically and that malin indeed localizes at glycogen, doing so via laforin. This mouse line should, in the future, help to identify malin's authentic glycogen-related substrate and novel glycogen metabolism pathway.

## RESULTS

### FLAG-malin mice do not form LBs

**Fig. 1.**
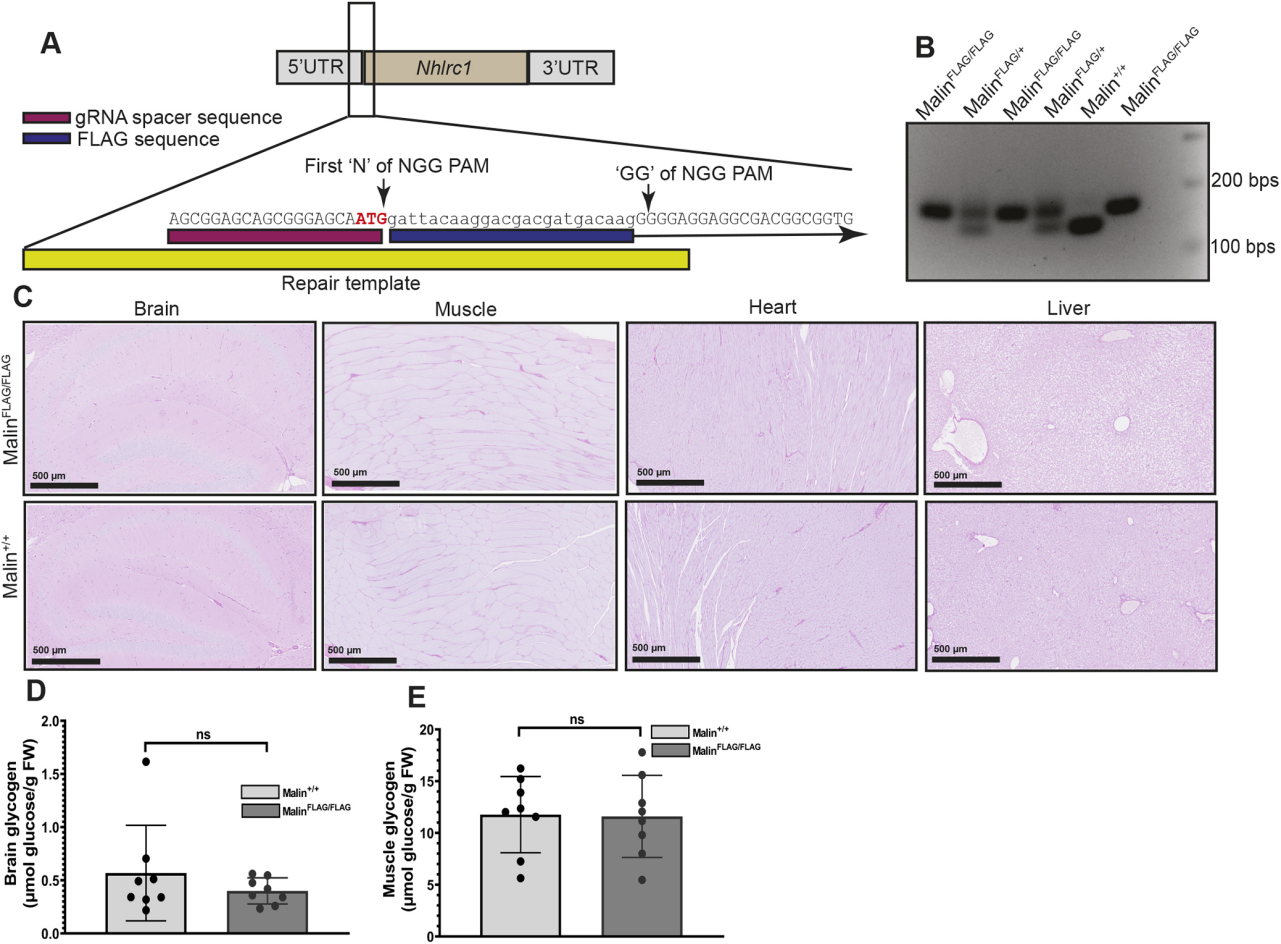
**Creation of a FLAG-malin mouse model to study malin protein.** (A) Schematic of the *Nhlrc1* exon, gRNA spacer (magenta), FLAG sequence (blue) and repair template (yellow). The first ‘N’ and ‘GG’ of the protospacer adjacent motif (PAM) sequence after the FLAG tag insertion are indicated by arrows. The start codon is bold and highlighted in red. (B) Genotyping results of mice obtained from FLAG-malin heterozygous–heterozygous intercross. Note that addition of 25 nucleotide FLAG sequence results in a PCR band shift in Malin^FLAG/FLAG^ mice. (C) Representative images from periodic acid–Schiff diastase (PAS-D)-stained brain, muscle, heart and liver from 12-month-old Malin^FLAG/FLAG^ and Malin^+/+^ mice are shown. Images represent *n*=6 per group with equally distributed males and females. (D,E) Glycogen measurements from 3-month-old homozygous FLAG-malin mice (Malin^FLAG/FLAG^) and their WT littermates (Malin^+/+^) from brain (D) and muscle (E). Males and females are equally distributed in each group with *n*=8. Data are means±s.d. ns, not significant (unpaired two-tailed Student's *t*-test). FW, fresh weight of the tissues.

**Fig. 2.**
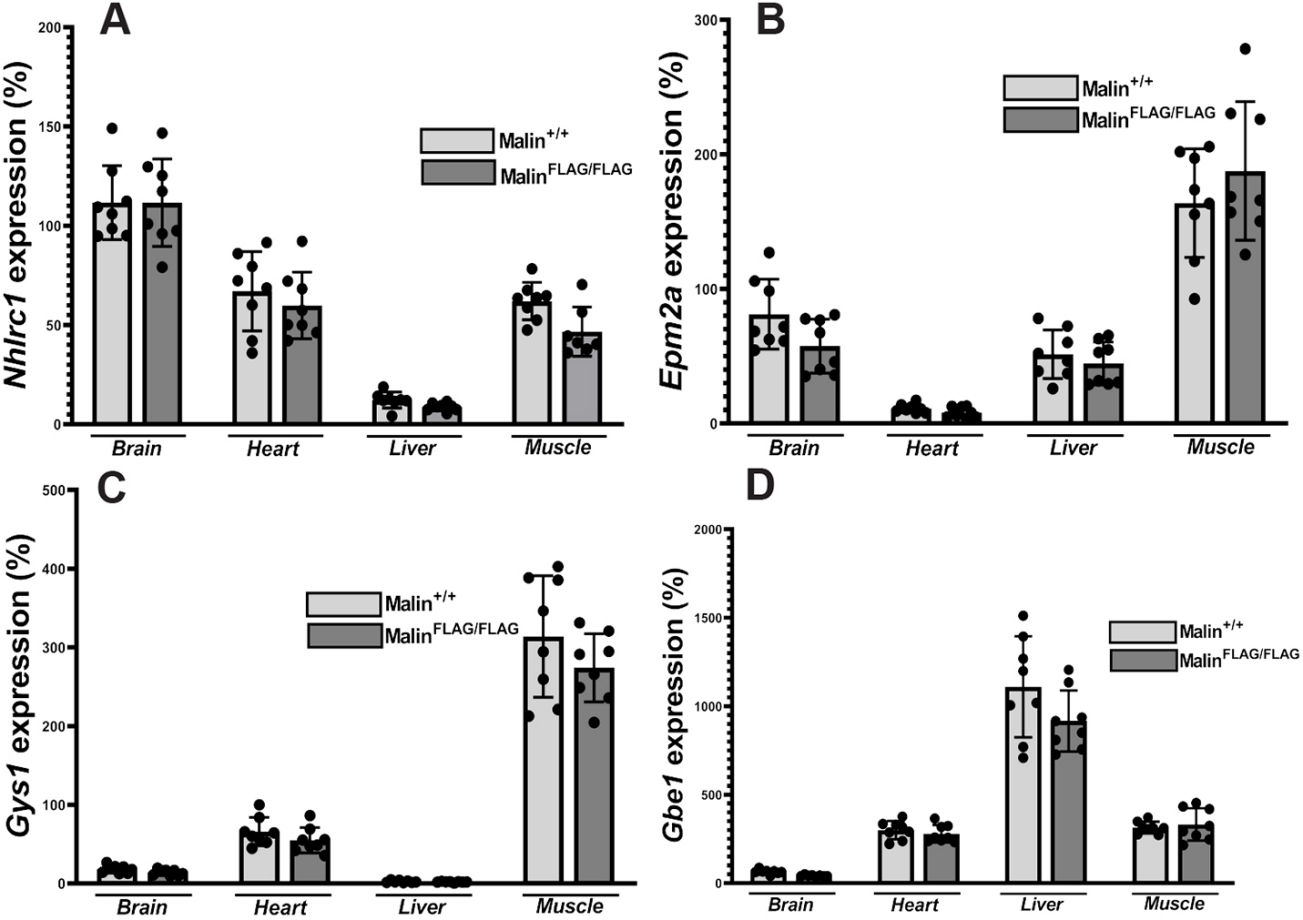
**Quantitative PCR shows physiological expression of *Nhlrc1* in four tissues from FLAG-malin mice, with no alterations in expression of *Epm2a, Gys1* (glycogen synthase) and *Gbe1* (glycogen branching enzyme).** (A-D) Total RNA from indicated tissues (3 months, males and females equally distributed with *n*=8) were extracted and analyzed for quantification of expression of the indicated genes. Values are expressed as means±s.d.

Loss of malin function results in LB formation in different organs – a hallmark of LD (Mitra et al., 2022). To determine whether insertion of the FLAG tag inactivated malin, we tested whether aged FLAG-malin mice form LBs. At 12 months, homozygous FLAG-malin mice (Malin^FLAG/FLAG^) exhibited no LBs in any organs tested including the brain (Fig. 1C). LBs are accumulations of glycogen, albeit malstructured, and Malin^−/−^ animals therefore exhibit progressive increase in glycogen quantities with the gradual expansion of the LB footprint (Criado et al., 2012; DePaoli-Roach et al., 2010; Turnbull et al., 2010; Valles-Ortega et al., 2011; Varea et al., 2022). Total glycogen quantities in brain and muscle in Malin^FLAG/FLAG^ mice were the same as those in wild-type (WT; Malin^+/+^) littermates (Fig. 1D,E; Fig. S2A). Finally, to rule out an effect of the FLAG insertion on expression of *Nhlrc1* or other glycogen metabolism genes, we performed quantitative real-time PCR (qPCR) on cDNA from several tissues and tested expression profiles of multiple glycogen metabolism-related genes. For all genes tested, including *Nhlrc1*, expression levels were the same between Malin^FLAG/FLAG^ and Malin^+/+^ (Fig. 2A-D; Fig. S1A-G). These results indicate that insertion of the FLAG tag does not affect expression of *Nhlrc1* or other glycogen metabolism genes and does not alter malin function to any relevant degree.

### FLAG-malin associates with glycogen

This and the next section will benefit from clarifying what we mean by the low-speed supernatant (LSS), low-speed pellet (LSP) and high-speed pellet (HSP) fractions of tissue extracts (details are provided in the Materials and Methods). Whole-tissue extract is first separated into the LSS and LSP by centrifugation on the benchtop at 8000 ***g***. This removes non-homogenized organelles and cell debris into the LSP. The HSP is the portion of the LSS that pellets out with ultracentrifugation at 100,000 ***g***. It contains soluble cytosolic macromolecules, principally (although not exclusively) glycogen (DePaoli-Roach et al., 2010; Tagliabracci et al., 2008). We would also like to state that because our initial characterization of the FLAG-malin mouse model using glycogen measurement, qPCR and periodic acid–Schiff diastase (PAS-D) staining (Fig. 1) did not reveal any difference between males and females, in the subsequent biochemical experiments we showed only male data, indicating cases in which we studied females and no differences were found.

**Fig. 3.**
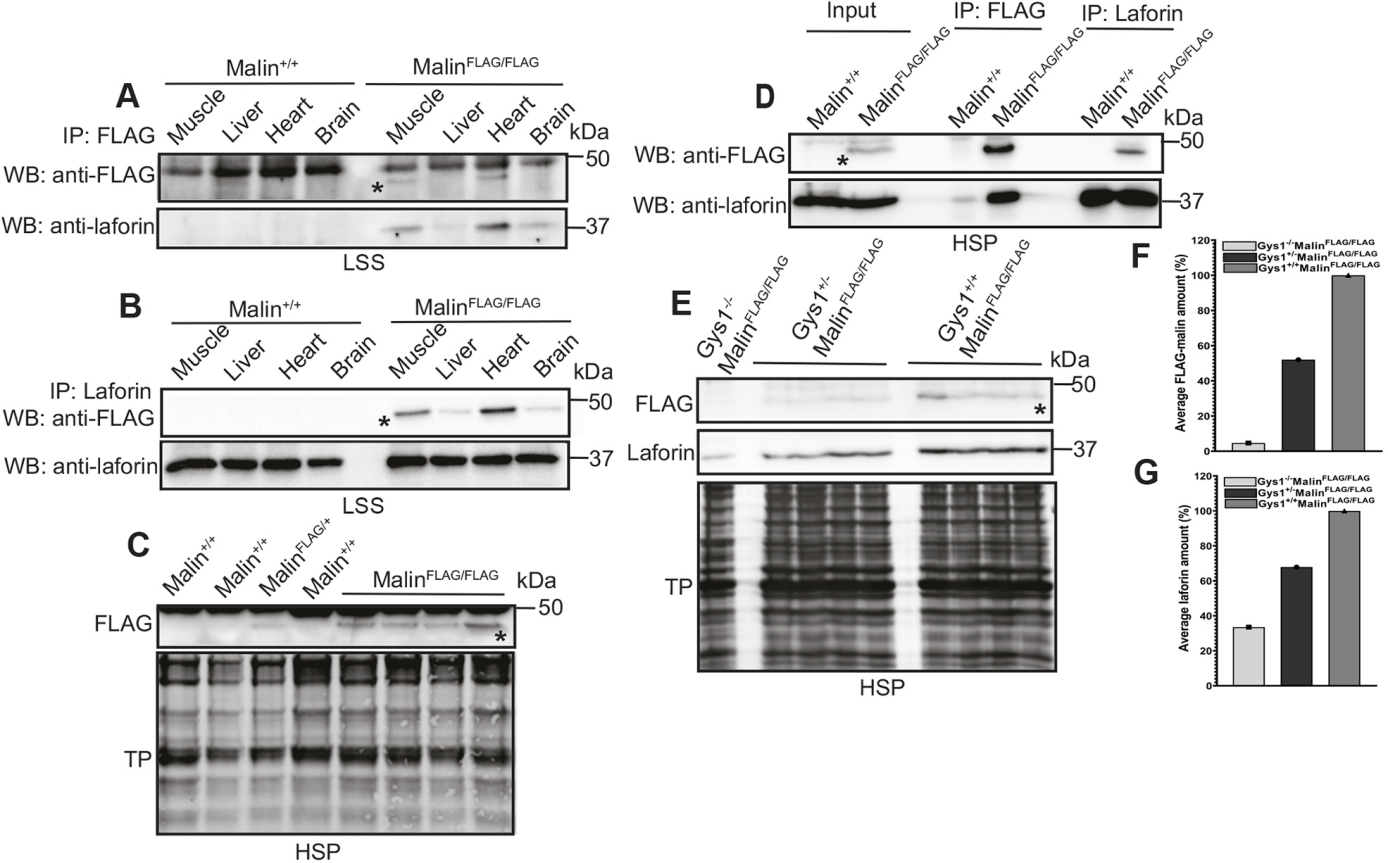
**Malin associates with soluble glycogen where it interacts with laforin.** (A,B) Co-immunoprecipitation (IP) of FLAG-malin (A) and laforin (B) in multiple tissues. Lysates from the indicated tissues of male Malin^FLAG/FLAG^ and Malin^+/+^ mice were immunoprecipitated using anti-FLAG or anti-laforin antibodies, and immunoblot analysis was carried out using anti-FLAG (upper blot) or anti-laforin (lower blot) antibodies. In A and B, representative images from two independent experiments are shown. Similar results were obtained with female mice (Fig. S4A). (C) Malin is found in the high-speed pellet (HSP) fraction. Immunoblot analysis using anti-FLAG with HSP fraction from muscle tissues from the indicated genotypes of male mice shows FLAG-malin band in Malin^FLAG/+^ or Malin^FLAG/FLAG^ mice but not in Malin^+/+^ mice (upper blot). Lower blot shows total protein (TP). Similar results were obtained with female mice (Fig. S4B). (D) Co-IP of FLAG-malin and laforin at the HSP fraction. HSP fractions from muscle tissues of male Malin^FLAG/FLAG^ and Malin^+/+^ mice were immunoprecipitated using anti-FLAG or anti-laforin antibodies, and the HSP fraction (input) or the immunoprecipitates were immunoblotted with anti-FLAG (upper blot) and anti-laforin (lower blot) antibodies. Similar results were obtained with female mice (Fig. S4C). (E) Within HSP fractions, malin associates with soluble glycogen. Immunoblot analysis using anti-FLAG and anti-laforin with HSP fraction from muscle tissues of the indicated genotypes of male mice shows reduced amount of FLAG-malin (upper blot) in Gys1^+/−^Malin^FLAG/FLAG^ mice compared with Gys1^+/+^Malin^FLAG/FLAG^ mice. The FLAG-malin band was almost undetectable in Gys1^−/−^Malin^FLAG/FLAG^ mice. Laforin was also reduced but detectable in both Gys1^+/−^Malin^FLAG/FLAG^ and Gys1^−/−^Malin^FLAG/FLAG^ mice (middle blot). Lower blot shows TP. (F,G) Quantification of E, showing average FLAG-malin (F) and laforin (G) amount in Gys1^+/−^Malin^FLAG/FLAG^ mice (average is calculated from *n*=4) and Gys1^+/+^Malin^FLAG/FLAG^ mice (average is calculated from *n*=4). For Gys1^−/−^Malin^FLAG/FLAG^, only one mouse was used due to difficulties in obtaining such a genotype (Pederson et al., 2004). In all blots, FLAG-malin band is indicated by an asterisk. LSS, low-speed supernatant; WB, western blotting.

It has, to date, not been possible to detect native malin in murine tissue extracts, either by western blotting or immunoprecipitation (IP). In the present work, multiple attempts at western blotting against the FLAG epitope of FLAG-malin in LSS fractions of tissue extracts from the FLAG-malin mice failed (Fig. S2B). However, IP with the FLAG antibody succeeded; IP from LSS fractions of skeletal and cardiac muscle specifically identified FLAG-malin in these two tissues. IP from LSS fractions of liver and brain did not (Fig. 3A, upper blot).

The second LD gene product, laforin, has long been suspected to be a malin interacting partner based on theoretical grounds and overexpression experiments (Gentry et al., 2005; Lohi et al., 2005; Solaz-Fuster et al., 2008; Worby et al., 2008). Now that we were able to immunoprecipitate FLAG-malin from tissues expressing it in normal malin amounts, we tested whether laforin precipitates. Indeed, laforin co-precipitated with FLAG-malin from LSS fractions of skeletal and cardiac muscle (Fig. 3A, lower blot). Remarkably, it also co-precipitated with FLAG-malin from LSS fractions of liver and brain. This suggests that IP of FLAG-malin pulls this protein down in all four tissues, with subsequent western blotting able to identify it only in the muscle extracts, while detecting the co-precipitated interactor laforin in all four tissues. We furthermore performed a reverse co-IP to confirm the interaction between laforin and malin in all the tissues indicated above. Here, we immunoprecipitated laforin from LSS fractions using an anti-laforin antibody, which pulled down both FLAG-malin (Fig. 3B, upper blot) and laforin (Fig. 3B, lower blot) in all indicated tissues including brain and liver. This substantiates our previous observation that FLAG-malin interacts with laforin in all four tissues.

We next hypothesized that FLAG-malin is localized on glycogen, which represents a small fraction of total tissue LSS, contributing to the difficulty in detecting the protein using whole LSS. We therefore proceeded to enrich for the glycogen portion of skeletal muscle LSS by isolating the glycogen-rich HSP portion. Western blotting for FLAG in the HSP allowed direct detection of FLAG-malin in that fraction (Fig. 3C). IP of FLAG-malin from the HSP pulled down FLAG-malin and laforin, suggesting that the two proteins associate at glycogen (Fig. 3D). We were not able to detect FLAG-malin by immunoblotting brain HSP fraction (Fig. S2C) or the LSP fractions from different tissues (Fig. S2D).

As mentioned, the HSP is composed predominantly of glycogen, but not exclusively. To confirm that FLAG-malin associates with glycogen in the HSP, we repeated the above experiments using skeletal muscle from Gys1^+/−^Malin^FLAG/FLAG^ and Gys1^−/−^Malin^FLAG/FLAG^ mice, which should have glycogen levels ∼50% and 0% of normal (Pederson et al., 2004), respectively. In Gys1^+/−^Malin^FLAG/FLAG^ tissue, the association of FLAG-malin with glycogen in the HSP fraction was reduced by ∼50%, and in Gys1^−/−^Malin^FLAG/FLAG^ tissue it was essentially eliminated (∼95% reduced; Fig. 3E,F). Laforin levels were also reduced in HSP fractions in these genotypes by ∼33% in Gys1^+/−^Malin^FLAG/FLAG^ and 67% in Gys1^−/−^Malin^FLAG/FLAG^ (Fig. 3E,G). These results show that malin associates with glycogen where it also interacts with laforin.

### FLAG-malin's association with glycogen is laforin dependent

**Fig. 4.**
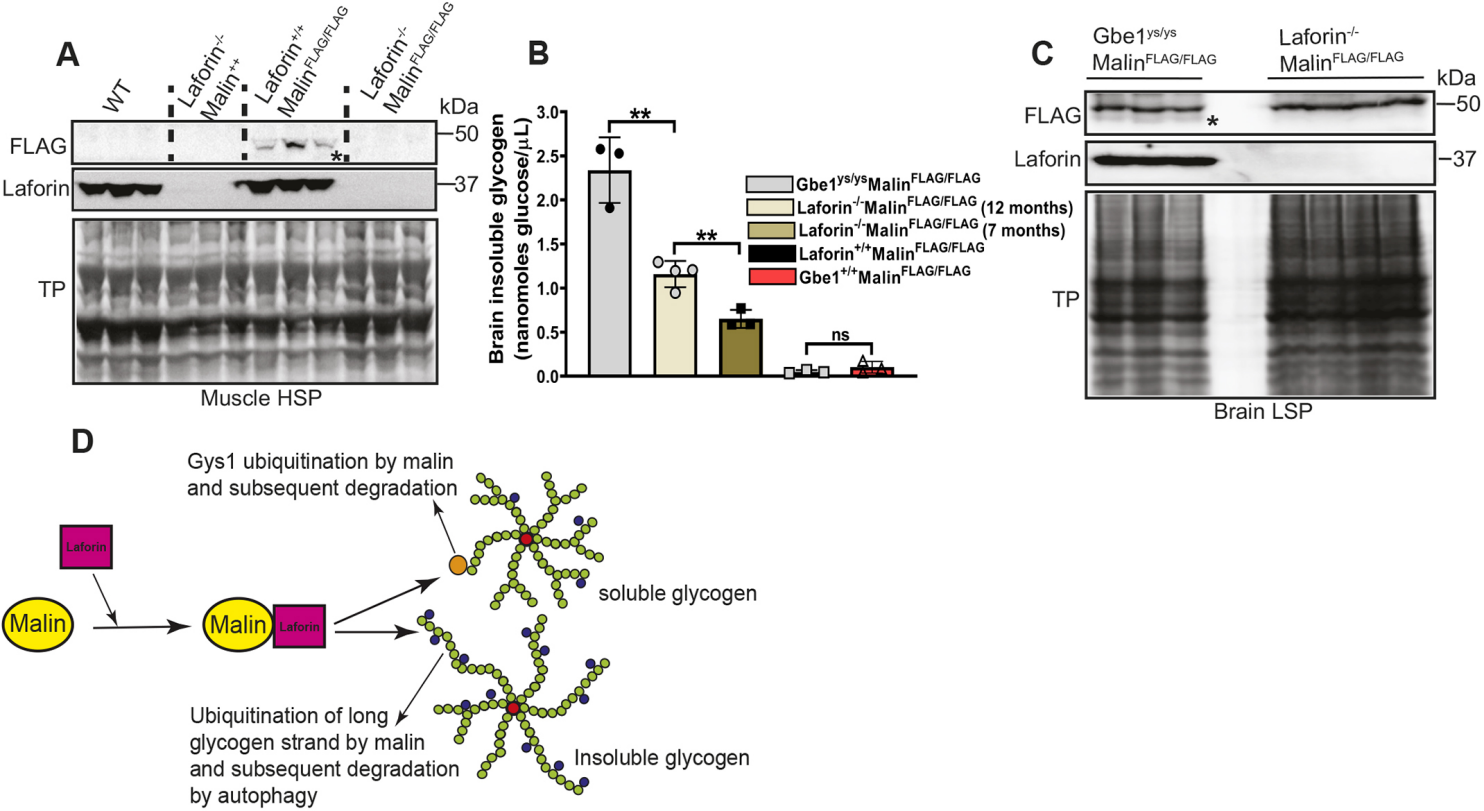
**Malin associates with soluble and insoluble glycogen only when laforin is present.** (A) Malin association with soluble glycogen depends on laforin. Immunoblot analysis using anti-FLAG in HSP fractions from muscle tissues of the indicated genotypes of male mice shows FLAG-malin bands in Laforin^+/+^Malin^FLAG/FLAG^ mice but not in Laforin^−/−^Malin^FLAG/FLAG^, Laforin^−/−^Malin^+/+^ or Laforin^+/+^Malin^+/+^ [wild-type (WT)] mice (upper blot). Middle blot shows laforin bands using anti-laforin antibody in Laforin^+/+^Malin^FLAG/FLAG^ and Laforin^+/+^Malin^+/+^ (WT) mice but not in Laforin^−/−^Malin^FLAG/FLAG^ or Laforin^−/−^Malin^+/+^ mice. Lower blot shows total protein (TP). Similar results were obtained when female mice were used (see Fig. S3A). (B,C) In brain, malin associates with polyglucosan bodies in a laforin-dependent manner. (B) Measurement of insoluble glycogen from brain low-speed pellets (LSPs) of the indicated genotypes of male mice. Seven-month-old Gbe1^ys/ys^Malin^FLAG/FLAG^ mice had the highest amount of glucose released per microliter of LSP homogenate (i.e. the largest amount of polyglucosan accumulation) followed by 12-month-old and 7-month-old Laforin^−/−^Malin^FLAG/FLAG^ mice. As expected, 7-month-old Gbe1^+/+^Malin^FLAG/FLAG^ and 12-month-old Laforin^+/+^Malin^FLAG/FLAG^ mice released negligible amounts of glucose from the LSP fraction (because they do not accumulate polyglucosans). Three to four mice per genotype were used. Data are shown as means±s.d. **P<0.01; ns, not significant (unpaired two-tailed Student's *t*-test). (C) Immunoblot analysis using anti-FLAG in LSP fractions from brain tissues of the indicated genotypes of male mice shows FLAG-malin bands in Gbe1^ys/ys^Malin^FLAG/FLAG^ mice but not in Laforin^−/−^Malin^FLAG/FLAG^ mice (upper blot). Middle blot shows that laforin is detectable by immunoblotting in Gbe1^ys/ys^Malin^FLAG/FLAG^ mice but not in Laforin^−/−^Malin^FLAG/FLAG^ mice. Lower blot shows TP. In this experiment, protein loading in the gel is normalized to the corresponding glycogen amount in the LSP as described in the Materials and Methods. (D) A model of the possible implication of malin association with glycogen via laforin. In cytosol, malin forms a complex with laforin, which targets it to soluble glycogen or insoluble glycogen. At glycogen, the laforin–malin complex acts as a checkpoint to ubiquitinate and degrade glycogen metabolism-related enzymes responsible for glycogen chain elongation (Gys1) and salvage precipitation-prone glycogen, and/or directly ubiquitinate insoluble glycogen to degrade it via autophagy. In all blots, FLAG-malin band is indicated by an asterisk.

Laforin possesses a typical carbohydrate-binding module of the CBM20 class found in three known mammalian proteins and countless others in the plant and other kingdoms (Gentry et al., 2013). Malin does not have a recognizable carbohydrate-binding domain. Meanwhile, as mentioned, laforin was recently shown to scaffold other proteins (Mst1/Mst2) to glycogen (Liu et al., 2021). We therefore reasoned that laforin may likewise scaffold malin to glycogen, and that malin's presence at glycogen is laforin dependent. We tested this in Laforin^−/−^Malin^FLAG/FLAG^ mice and controls. Immunoblotting for FLAG-malin in skeletal muscle HSP showed the presence of FLAG-malin only when laforin was present (Fig. 4A; Fig. S3A), confirming that malin is targeted to glycogen by laforin.

### FLAG-malin associates with polyglucosan bodies via laforin

Our knowledge of how the laforin–malin complex regulates glycogen structure to ensure that it has short branches that allow it to remain soluble despite its large size (∼55,000 glucose units) ends at malin, because malin's relevant E3 ligase substrate remains unknown. Here, we asked whether malin associates with the insoluble polyglucosans (LBs) in laforin knockout LD mice. Being insoluble, LBs segregate with the LSP fraction (Sullivan et al., 2019). Western blotting for FLAG-malin in the brain LSP of Laforin^−/−^Malin^FLAG/FLAG^ revealed the absence of FLAG-malin. To be able to interpret this negative result in subsequent experiments, we included an additional genotype we hoped would serve as a positive control. Polyglucosans form in a few diseases other than LD, including adult polyglucosan body disease (APBD), caused by deficiency of glycogen branching (*GBE1*) (Robitaille et al., 1980). The most common APBD mutation is Y329S, for which there is a mouse model (Gbe1^ys/ys^) (Akman et al., 2011; Lossos et al., 1998; Orhan Akman et al., 2015). We generated Gbe1^ys/ys^Malin^FLAG/FLAG^ mice, obtained their brain LSP fractions, and included them with brain LSP fractions from Laforin^−/−^Malin^FLAG/FLAG^ mice in the same gel. Loading was based on equal polyglucosan amounts (20 nmol glucose; Fig. 4B). Again, the Laforin^−/−^Malin^FLAG/FLAG^ LSP showed no evidence of presence of FLAG-malin, but the Gbe1^ys/ys^Malin^FLAG/FLAG^ LSP did (Fig. 4C). These results indicate that malin does, or can, localize to polyglucosans, but seemingly only when its laforin partner is present.

## DISCUSSION

LD was described in 1911, and its disease genes were reported in 1998 (*EPM2A*/laforin) and 2003 (*Nhlrc1*/malin) (Chan et al., 2003; Lafora and Glueck, 1911; Minassian et al., 1998). As mentioned, how laforin and malin regulate glycogen structure is unknown. With the identification of laforin's phosphatase activity and demonstration that it dephosphorylates glycogen, the strong possibility emerged that glycogen over phosphorylation underlies polyglucosan generation (Ganesh et al., 2000; Minassian et al., 1998; Tagliabracci et al., 2008; Worby et al., 2006); however, subsequent work showed that inactivation of laforin's phosphatase, although it leads to glycogen overphosphorylation, is not sufficient to lead to polyglucosan generation or LBs (Gayarre et al., 2014; Nitschke et al., 2017). Instead, laforin or malin mutations that disrupt laforin binding to glycogen or to malin, or that inactivate malin's ubiquitin ligase activity, all cause LD (Brewer et al., 2021), indicating that a malin function at glycogen is critical to ensuring glycogen structural integrity. Despite being known to play a crucial role in glycogen metabolism and LD for 20 years, what malin's exact role is has remained unknown in large part because the protein could, until now, not be detected in mouse tissues. Malin could also not be studied in lower vertebrates and other species, because a confirmed ortholog is not present e.g. in zebrafish, *Drosophila*, nematodes or yeast (Romá-Mateo et al., 2011). In the present work, we tagged *Nhlrc1* at its native locus, and demonstrated that this does not affect gene expression and does not impact protein function, at least not to any degree of significance to LD. Our mouse model permits detection of malin in tissues by IP and in its apparent major locale, glycogen, by direct western blotting. This model should help to initiate resumption of the search for malin's authentic substrate that regulates cytosolic glycogen structure and answers to subsequent questions arising.

One of the new findings in our study was malin association with glycogen. This was shown by pulling down soluble muscle glycogen, followed by detection of the malin protein by biochemical means. Compared to brain, muscle is a glycogen-enriched tissue (0.1% of total brain weight versus 1-2% of muscle mass (Brown, 2004; Nelson et al., 1968; Shulman et al., 1995). Additionally, adult mouse brain glycogen is known to be depleted as much as 46% within a minute of post-euthanasia by routine laboratory techniques such as cervical dislocation or decapitation before rapid freezing (DiNuzzo et al., 2019; Hutchins and Rogers, 1970; Juras et al., 2022 preprint). Consequently, our attempt to track malin in brain HSP fraction failed. Additionally, LSP fractions from WT and FLAG-malin mice did not show any FLAG-malin band, although its interacting partner laforin was present. Consistently, HSP fractions from mice with reduced or no glycogen synthase showed negligible amounts of FLAG-malin compared to mice expressing the enzyme. The amount of laforin in the HSP fractions was also reduced in these mice. Considering all the above, it is evident that, within cells, malin's primary locale is soluble glycogen.

Our mouse model has allowed us to demonstrate that malin localizes at glycogen, to confirm that malin and laforin interact under physiological conditions, and to show that they do so at muscle glycogen. Furthermore, the model showed that laforin scaffolds malin to glycogen, without which malin cannot localize to the macromolecule for which it regulates structure. LBs are preponderantly polyglucosan, but because they are ever-enlarging aggregates they also trap and have been shown to contain a large variety of proteins (Augé et al., 2018; Ganesh et al., 2002; Puri et al., 2012; Rao et al., 2010; Riba et al., 2019; Thomsen et al., 2022). Remarkably, we find no evidence of malin (FLAG-malin) in LBs of laforin knockout LD mice, but the protein is present in polyglucosan bodies caused by a separate genotype (glycogen branching enzyme deficiency), suggesting that malin cannot even be nonspecifically trapped in polyglucosan masses without the presence of laforin, appearing to reinforce the criticality of laforin's scaffolding role to malin's glycogen function.

A serendipitous observation in our study is a side point not yet raised, relating to *Nhlrc1* brain expression level. *Nhlrc1* is a single-exon gene. Most such genes are expressed at low levels and more often have functions related to the nervous system (Aviña-Padilla et al., 2021; Brinster et al., 1988; Buchman and Berg, 1988; Louhichi et al., 2011; Shabalina et al., 2010). Fig. 2A shows that FLAG-*Nhlrc1* (and *Nhlrc1*) are expressed at high levels in the brain, substantially higher than in the major glycogen metabolizing organs: skeletal muscle, heart and liver. Whether this has neurobiological significance awaits to be seen, but at least suggests a critical role for malin in brain glycogen metabolism, consistent with the severity of the neurological disease resulting from its absence. *Epm2a* had highest expression level in muscle. This complementary expression of the two genes could explain the importance of laforin in stabilizing malin's cellular level (Mittal et al., 2015, 2018) or glycogen association as seen in our current study. It is possible that the higher amount of laforin in skeletal muscle compared to brain could augment malin's glycogen association in muscle but not in brain, as seen in our study. Furthermore, it could be possible that by complementing their expression at the RNA level, the two proteins are able to express in different tissues in the amount suitable to form a complex with perfect stoichiometry, ensuring their functions. Additionally, differential tissue expression of *Nhlrc1* and *Epm2a* could underlie certain diverse functions in different tissues or subcellular locations. For example, malin was recently shown to translocate to the nucleus and promote glycogen degradation in the lung, with implications for lung cancer, whereas laforin was shown to sequester tumor suppressor proteins at glycogen in the liver, with impacts on liver cancer (Liu et al., 2021; Sun et al., 2019).

Ultimately, the critical structural defect in polyglucosans is their overlong chains (Sullivan et al., 2019). The following are among the hypotheses that can be envisioned on how the laforin–malin complex regulates cytosolic glycogen chain lengths (Fig. 4D). (1) It is possible that laforin monitors for occasional imbalances between glycogen synthase and branching enzyme with the former outpacing the latter and generating localized overly long chains that drive affected molecules to precipitate. Laforin would bind such regions and recruit malin, and malin would act on one or more glycogen-metabolizing enzymes to limit the damage, e.g. to halt glycogen synthase or activate the chain-shortening enzyme glycogen phosphorylase. Hypotheses along this line suggest that laforin–malin plays a protective role in preventing polyglucosan formation. (2) Alternatively, laforin may bind already precipitated polyglucosans and attract malin, the protein substrate of which might activate mechanisms to clear polyglucosans before they aggregate into toxic masses. Such mechanisms might include autophagy, as previously suggested (Sánchez-Martín et al., 2015). Autophagy could be activated by malin through ubiquitination of autophagy pathway proteins (Ganesh et al., 2002; Puri et al., 2012; Sánchez-Martín et al., 2020, 2015) or even, possibly, through direct ubiquitination of the polyglucosans themselves. The latter possibility is supported by three observations. (1) Malin's partner laforin, uniquely among all known mammalian phosphatases, acts directly on (dephosphorylates) glycogen. (2) A small portion of glucose units in glycogen are aminated. This fraction reaches high amounts in brain glycogen (up to 20% of brain glycogen glucose is actually glucosamine), and even larger amounts in polyglucosans (Sun et al., 2021). These amines could serve as receiving moieties in the putative glycogen ubiquitinating reaction. (3) Recently, another E3 ubiquitin ligase, RBCK1, was shown to ubiquitinate glycogen and even more so long-chained glucans *in vitro* (Kelsall et al., 2022). Loss of RBCK1 function results in polyglucosan accumulation and a distinct disease, characterized by fatal skeletal and cardiac myopathy (AlAnzi et al., 2022; Boisson et al., 2012; Chen et al., 2021; Nilsson et al., 2013; Phadke et al., 2020; Wang et al., 2013). Hypotheses along these lines suggest that the laforin–malin complex functions in actively clearing polyglucosans.

There is no treatment for LD, and the suffering of affected patients and families continues unabated. It is hoped that a more complete understanding of laforin–malin function will illuminate possible targets for therapeutic intervention. Glycogen synthesis has long been considered settled science, where glycogen synthase and branching enzyme act in concert to generate perfectly short-branched molecules (Roach et al., 2012). LD shows us that there is more to discover, which may impact much more common conditions related to defects in glycogen metabolism. For example, it has long been known that an unknown defect in glycogen synthesis plays an important, but not understood, role in insulin resistance (Shulman et al., 1990; Thorburn et al., 1990). A more complete picture of glycogen synthesis, possibly facilitated by the present FLAG-malin model, may also contribute to this area of research.

## MATERIALS AND METHODS

### Animals

#### FLAG-malin mouse

The FLAG-malin mouse model was generated at The Centre for Phenogenomics (TCP), Toronto, Canada, with procedures compliant with the Animals for Research Act of Ontario and the Guidelines of the Canadian Council on Animal Care. The TCP Animal Care Committee reviewed and approved all procedures.

N-terminal insertion of the FLAG tag (gattacaaggacgacgatgacaag) into the *Nhlrc1* open reading frame was carried out using CRISPR/Cas9 engineering. The guide RNA (gRNA) was designed using CRISPOR (Haeussler et al., 2016) with specificity confirmed using Cas-OFFinder (Bae et al., 2014) and off-target protospacer adjacent motif (PAM) set to NRG (where N is A, T, C or G and R is G or A). The gRNA was synthesized from a gblock gene fragment (Integrated DNA Technologies) using a MEGAshortscript T7 Transcription Kit (Invitrogen, AM1354). It was purified with a MEGAclear™ Kit (Life Technologies, AM1908) and visualized on an agarose gel to assess integrity. A single-strand oligonucleotide repair template with 70 bp homology arms flanking the FLAG tag immediately downstream of the start codon was used (Fig. 1A). C57BL/6J (The Jackson Laboratory, 3- to 4-week-old females) mice were used as embryo donors and Crl:CD-1 (ICR) (Charles River Laboratories) outbred albino stock was used as pseudopregnant recipients. Donor females were superovulated by intraperitoneal injection of 5 IU of pregnant mare's serum gonadotropin (Prospec, HOR-272) followed 48 h later by an intraperitoneal injection of 5 IU human chorionic gonadotrophin (hCG; EMD Millipore, 230734) and mated overnight with proven breeder males. Oviducts were dissected at ∼22 h post hCG, and cumulus oocyte complexes were released in M2 medium (Cytospring) and treated with 0.3 mg/ml hyaluronidase (Sigma-Aldrich, H4272) as described (Behringer et al., 2014). Fertilized embryos were selected and kept at 37°C, 6% CO_2_ in microdrops of KSOM medium with amino acids (KSOM^AA^; Cytospring) covered by embryo-tested paraffin oil (Zenith Biotech, ZPOL-50) prior to pronuclear microinjection. For pronuclear microinjection, injection mixes were prepared as described (Gertsenstein and Nutter, 2018) and consisted of 20 ng/µl Cas9 mRNA (Life Technologies, A23978), 10 ng/µl gRNA (AGCGGAGCAGCGGGAGCAAT) and 10 ng/µl single-stranded repair template (ATGCCCCGCTCCGTGACCGTGACCGTGGCCGTGACTGAGGGCTGCGCGGAGGCAGCGGAGCAGCGGGAGCAATGgattacaaggacgacgatgacaagGGGGAGGAGGCGACGGCGGTGGCAGCGGCTGGGGTGCGGCCCGAGCTGGTGCGGGAGGCGGAGGTCAGCC) in microinjection buffer (10 mM Tris, 0.1 mM EDTA). Pronuclear micronjections into zygotes were performed as described (Behringer et al., 2014). Briefly, gRNA/Cas9 mixture was injected into the pronuclei of ∼100 zygotes using glass capillaries with inner filament manufactured in-house or purchased from Biomedical Instruments with a tip diameter of ∼1.2 µm and Eppendorf FemtoJet 4i set at constant flow. Micronjections were performed under 200-250× magnification on a Leica inverted microscope with Nomarski differential interference contrast using Leica micromanipulators. Embryos were briefly cultured in KSOM^AA^ and transferred into the oviducts of 0.5 days post coitum pseudopregnant CD-1 (ICR) female recipients shortly after manipulations.

Tail tissue samples were collected from 14-day-old putative founder pups, and genomic DNA was isolated using an Extracta DNA prep kit (Quanta Biosciences, 95091-025) following the manufacturer's protocol. Sequencing of PCR amplicons revealed evidence of tag insertion in three of the 12 mice derived from the micro-injected embryos. These mice were bred with C57BL/6J mice to generate N1 mice. PCR amplicons from these mice were then subcloned into a vector, transformed and sequenced from within the 5′ untranslated region (UTR) to within the 3′ UTR of the *Nhlrc1* gene. To verify that the repair template was not inserted elsewhere in the genome, qPCR was conducted using a probe (AGCGGAGCAGCGGGAGCAAT) with 5′ FAM dye/3′ Zen/IABkFQ double quencher (Integrated DNA Technologies), forward primer (5′-GTCGGCCGTGACTGAGG-3′) and reverse primer (5′-CTCCCGCACCAGCTC-3′). The assay detects WT sequences within the repair template with DNA from a WT mouse serving as a two-copy control. As published earlier (Gertsenstein and Nutter, 2018), the TCP core considered target copy numbers ranging from 1.8 to 2.2 for autosomal genes in males as WT copy. qPCR confirmed no exogenous insertions of the repair template in the FLAG-malin mouse.

The N1 mouse with perfect sequence and a copy number of 2.16 was further bred with C57BL/6J to obtain heterozygous FLAG-malin (Malin^FLAG/+^) mice. Intercross among Malin^FLAG/+^ male and female generated the desired homozygous FLAG-malin (Malin^FLAG/FLAG^) mice and their WT littermates (Malin^+/+^).

#### FLAG-malin-laforin knockout mouse

The laforin knockout mouse model used in this study has been described previously (Ganesh et al., 2002). Mice heterozygous for FLAG-malin and laforin knockout mice (Malin^FLAG/+^ and Laforin^+/−^) were crossed with each other to generate mice heterozygous for both (Laforin^+/−^Malin^FLAG/+^). Males and females of these mice were crossed to generate laforin knockout mice with homozygous FLAG-malin (Laforin^−/−^Malin^FLAG/FLAG^), WT laforin mice with homozygous FLAG-malin (Laforin^+/+^Malin^FLAG/FLAG^) and WT littermates (Laforin^+/+^/Malin^+/+^).

#### FLAG-malin-Gys1 knockout mouse

In order to create a FLAG malin mouse lacking one or both alleles of the glycogen synthase-1 (*Gys1*) gene, we used the Gys1 conditional-ready mouse published by us earlier (Nitschke et al., 2021). We crossed these mice with CAG-Cre mice (Sakai and Miyazaki, 1997) (a gift from Dr Philip Shaul, University of Texas Southwestern Medical Center) to create a mouse heterozygous for *Gys1* (Gys1^+/−^) due to excision of exons 6 to 8. Gys1^+/−^ mice were then crossed with homozygous FLAG-malin mice (Malin^FLAG/FLAG^) to obtain Gys1^+/−^Malin^FLAG/+^ mice. Finally, a cross among Gys1^+/−^Malin^FLAG/+^ mice created homozygous or heterozygous FLAG-malin mice with either complete (Gys1^−/−^Malin^FLAG/FLAG^) or partial (Gys1^+/−^Malin^FLAG/FLAG^) absence of the *Gys1* gene (Fig. S3B,C).

#### FLAG-malin-Gbe1 mutant mouse

Heterozygous mice for FLAG-malin and *Gbe1* (Malin^FLAG/+^ and Gbe1^ys/+^) were intercrossed with each other to generate a mouse heterozygous for both genes (Gbe1^ys/+^Malin^FLAG/+^). These were intercrossed to generate a Gbe1 mutant mouse with homozygous FLAG-malin (Gbe1^ys/ys^Malin^FLAG/FLAG^) or a WT Gbe1 mouse with homozygous FLAG-malin (Gbe1^+/+^Malin^FLAG/FLAG^) (Fig. S3D,E).

All experimental mice were housed at the University of Texas Southwestern Medical Center's Animal Resource Center facility. Mice were kept in ventilated cages with 12 h dark/light cycle at 25°C. They were fed standard laboratory chow and water *ad libitum*. Table S1 lists the genotyping primers used for FLAG-malin, CAG-Cre and *Gys1* knockout mice, and Fig. 1B shows a representative image of FLAG-malin mouse genotyping. Mice that were generated by intercrossing FLAG-malin mice with other strains were genotyped using previously published methods. For all biochemical experiments, mice were aged to appropriate months and sacrificed by cervical dislocation, and their tissues were snap frozen in liquid nitrogen and kept at −80°C until further use. For histological examination, mice were euthanized by cervical dislocation, and tissues were fixed in 10% neutral buffered formalin. Males and females were used in approximately equal numbers. All mouse procedures were approved by the Institutional Animal Care and Use Committee of the University of Texas Southwestern Medical Center.

### Glycogen measurement

Tissue glycogen content was measured as previously described (Gumusgoz et al., 2021; Nitschke et al., 2017). Briefly, total glycogen was extracted from frozen ground tissue by boiling in KOH and precipitating in ethanol/sodium sulfate. After three further rounds of precipitation in ethanol/LiCl, the glycogen-containing pellet was resuspended in sodium acetate and digested with amyloglucosidase (Megazyme) to liberate glucose. Glucose was then measured in both sample and blank digested controls enzymatically. The quantity of glucose was normalized to fresh weight to represent glucose derived from tissue glycogen. In order to measure the soluble glycogen from the HSP or the degradation-resistant glycogen (i.e. polyglucosans) from LSP homogenate, a 50 µl aliquot from the homogenate was taken. Glycogen or polyglucosans were then extracted, precipitated and measured following the same method described above.

### PAS-D staining

Paraffin-embedded tissues were sectioned followed by staining using PAS-D as described (Gumusgoz et al., 2021). A Hamamatsu Nanozoomer 2.0 HT digital slide scanner (40× objective) was used to scan stained slides.

### RNA extraction, cDNA synthesis and qPCR

Tissues were ground into powder using a method described previously (Gumusgoz et al., 2021; Sullivan et al., 2019), quickly transferred to TRiZol reagent (Invitrogen, 15596018) and homogenized using a BD-18G needle syringe. RNA was purified using a PureLink RNA Mini-Kit (Invitrogen, 12183018A) following the manufacturer's instructions. Purified RNA was quantified using a spectrophotometer, and a A260/280 ratio >2.0 indicated purity. Integrity and purity of the RNA samples were further visualized using agarose gel electrophoresis. One microgram of RNA from all indicated tissues was used to synthesize cDNA using iScript Reverse Transcription SuperMix (Bio-Rad, 1708841) following the manufacturer's instructions. qPCR was performed with 24 ng cDNA using iTag Universal SYBR Green Master Mix (Bio-Rad, 1725121) with a QuantStudio 7 Pro real-time cycler (Thermo Fisher Scientific). Target genes were normalized with three housekeeping genes, and data were compared with WT malin brain using the ΔΔCT method (Livak and Schmittgen, 2001; Rao et al., 2013). Primers used in the qPCR study are listed in Table S2.

### Immunoprecipitation and western blotting

Mouse brain, heart, liver and gastrocnemius muscles were lysed with ice-cold lysis buffer (10 mM Tris-HCl, pH 7.0, 150 mM KF, 0.6 M sucrose and 1.5 mM EDTA, 10 µl buffer/mg of tissue) supplemented with fresh protease inhibitor (Roche, 04693159001) and phosphatase inhibitor (Roche, 04906845001) cocktails. Tissues were dounced ∼40 times on ice, and the homogenate was spun at 8000 ***g*** for 10 min at 4°C to separate the LSS from the insoluble LSP. In some cases, LSS was subjected to ultracentrifugation at 100,000 ***g*** for 1.5 h at 4°C to obtain glycogen-enriched HSP. Next, the LSP and HSP were resuspended with lysis buffer by pipetting to homogeneity. An aliquot was taken from the LSS, LSP and HSP to measure protein concentration using a Bradford Assay kit (Thermo Fisher Scientific, 1863028). In order to immunoprecipitate proteins from the LSS or HSP, 700 µg of total proteins (in the case of the HSP, equivalent to ∼9-13 nmol released glucose from each sample) was mixed with either anti-FLAG antibody (OctA-Probe F-tag, Santa Cruz Biotechnology, sc-51590; 1:500) or anti-laforin antibody (Abnova, H00007957-D01; 1:100) and incubated overnight, gently rotating at 4°C. The next day, 20 µl of pre-cleaned protein G agarose beads (Cell Signaling Technology, 37478S) was added to each tube followed by incubation at 4°C for 4 h. Beads were then washed with ice-cold phosphate buffered saline (Thermo Fisher Scientific, BP399-4) with 0.1% Tween 20 (PBST). Bound materials were eluted by boiling in sample loading buffer and subjected to western blotting as described below.

For western blotting without IP, 40 µg protein (or where loading was based on equal amount of polyglucosans, micrograms of protein equivalent to 20 nmol released glucose from each LSP sample) was mixed with Laemmli sample buffer (Bio-Rad, 161-0747), boiled at 95°C and quickly centrifuged, and supernatant was loaded onto 10% Tris-glycine SDS gel. Resolved proteins were transferred to nitrocellulose membranes with Tris/glycine buffer (Bio-Rad, 1610771) supplemented with 20% methanol for 1.5 h with 350 mA. Immediately after transfer, total proteins were visualized using Revert 700 Total Protein Stain (LI-COR, 92611021) following the manufacturer's instructions. Membranes were blocked in 5% (w/v) milk powder (Bio-Rad, 706404) dissolved in PBST for 1 h at room temperature. Subsequently, membranes were probed with designated primary antibodies overnight at 4°C. The primary antibodies used in this study were mouse anti-laforin (Abnova, H00007957-M02, clone 6C6; 1:1000), mouse anti-FLAG (OctA-Probe F-tag, Santa Cruz Biotechnology, sc-51590; 1:500), rabbit anti-Gbe1 (Abcam, ab180596; 1:2500) and rabbit anti-Gys1 (Cell Signaling Technology, 15B1; 1:5000). The next day, membranes were washed in PBST followed by incubation with horseradish peroxidase (HRP)-coupled secondary antibody (HRP-conjugated goat anti-mouse IgG, light chain specific; Jackson ImmunoResearch, 115-035-174). For laforin, blots were incubated for 1 h at room temperature, while for FLAG-malin blots were incubated for 2 h at room temperature, followed by 3 h of incubation at 4°C. At the end, blots were washed in PBST and visualized using Immobilon Forte Western HRP Substrate (Millipore, WBLUF0500) on a ChemiDoc MP imager (Bio-Rad, 734BR-4002). Protein bands were quantified using Image Lab software (Bio-Rad). Finally, protein band intensity was normalized against the total protein lane.

### Statistical analysis

GraphPad Prism software (version 8.0.2) was used to generate all the graphs and perform the statistical analyses. Student's unpaired *t*-test (two-tailed) was used, and *P*<0.05 was considered significant.

## Supplementary Material

10.1242/dmm.049802_sup1 Supplementary information
