# Supplementary material for: Laforin targets malin to glycogen in Lafora progressive myoclonus epilepsy
Source: Dis Model Mech. 2023 Jan 6;16(1):dmm049802. doi: 10.1242/dmm.049802 (PMC9844227; doi:10.1242/dmm.049802)
Supplement: Supplementary information [file dmm-16-049802-s1.pdf]

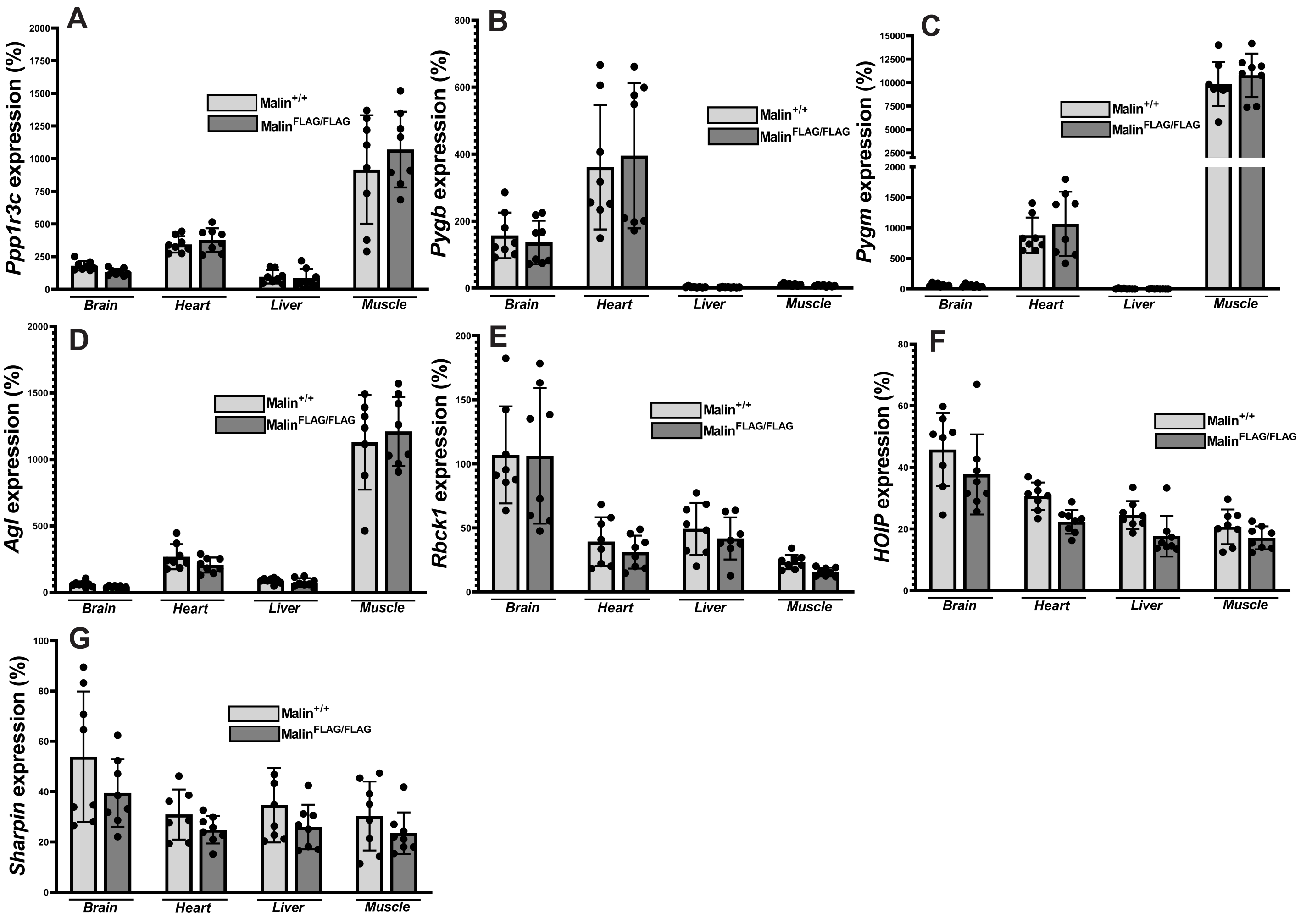

Fig. S1. Quantitative PCR reveals no alterations of expression of various glycogen metabolism related genes in FLAG malin mice. Total RNA from the indicated tissues (3 month-old, males and females are equally distributed with n=8) were extracted and analyzed for expression quantification of the expression of indicated genes. Panels A-D show classical genes involved in glycogen metabolism, whereas panels E-G show genes of components of the linear ubiquitin chain assembly complex (LUBAC), including RBCK1, more recently implicated in glycogen metabolism. Data are represented as means ± SD.

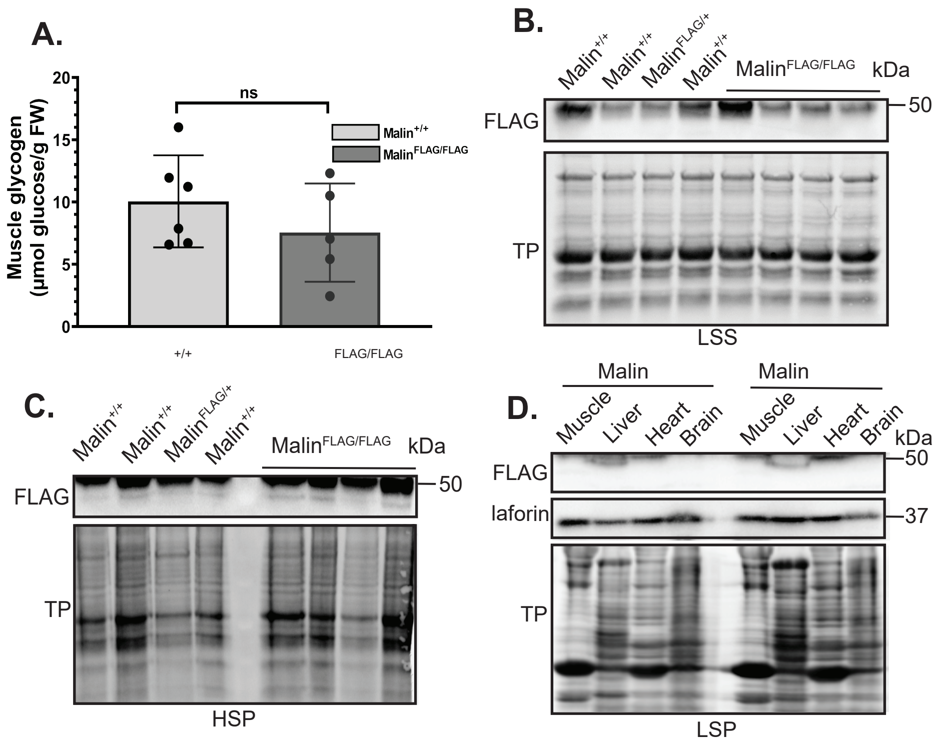

Fig. S2. Malin association with brain HSP and tissue LSP fractions. (A) Glycogen measurement from 7 month-old homozygous FLAG-malin mice (Malin$^{FLAG/FLAG}$) and their WT littermates (Malin$^{+/+}$) from muscle. Males and females are equally distributed in each group with n=5-6. Data are shown as means ± SD. Significance levels are: *, $P < 0.05$; **, $P < 0.01$; ***, $P < 0.001$; ****, $P < 0.0001$. FW, Fresh Weight of the tissues. (B) Immunoblot analysis using anti-FLAG with LSS fractions from muscle tissues of indicated genotypes of male mice shows no FLAG-malin band in Malin$^{FLAG/FLAG}$, Malin$^{FLAG/+}$ and Malin$^{+/+}$ mice. (C) Immunoblot analysis using anti-FLAG with HSP fractions from brain tissues of indicated genotypes of male mice (n=4) shows no FLAG-malin band in both Malin$^{FLAG/FLAG}$ and Malin$^{+/+}$ mice. (D) Immunoblot analysis using anti-FLAG with LSP fractions from indicated tissues of male Malin$^{FLAG/FLAG}$ and Malin$^{+/+}$ mice shows no FLAG-malin band (upper blot). The same blot was stripped and probed for laforin using an anti-laforin antibody, which showed the protein band (middle blot). In B-D, the lower blot is for total protein (TP).

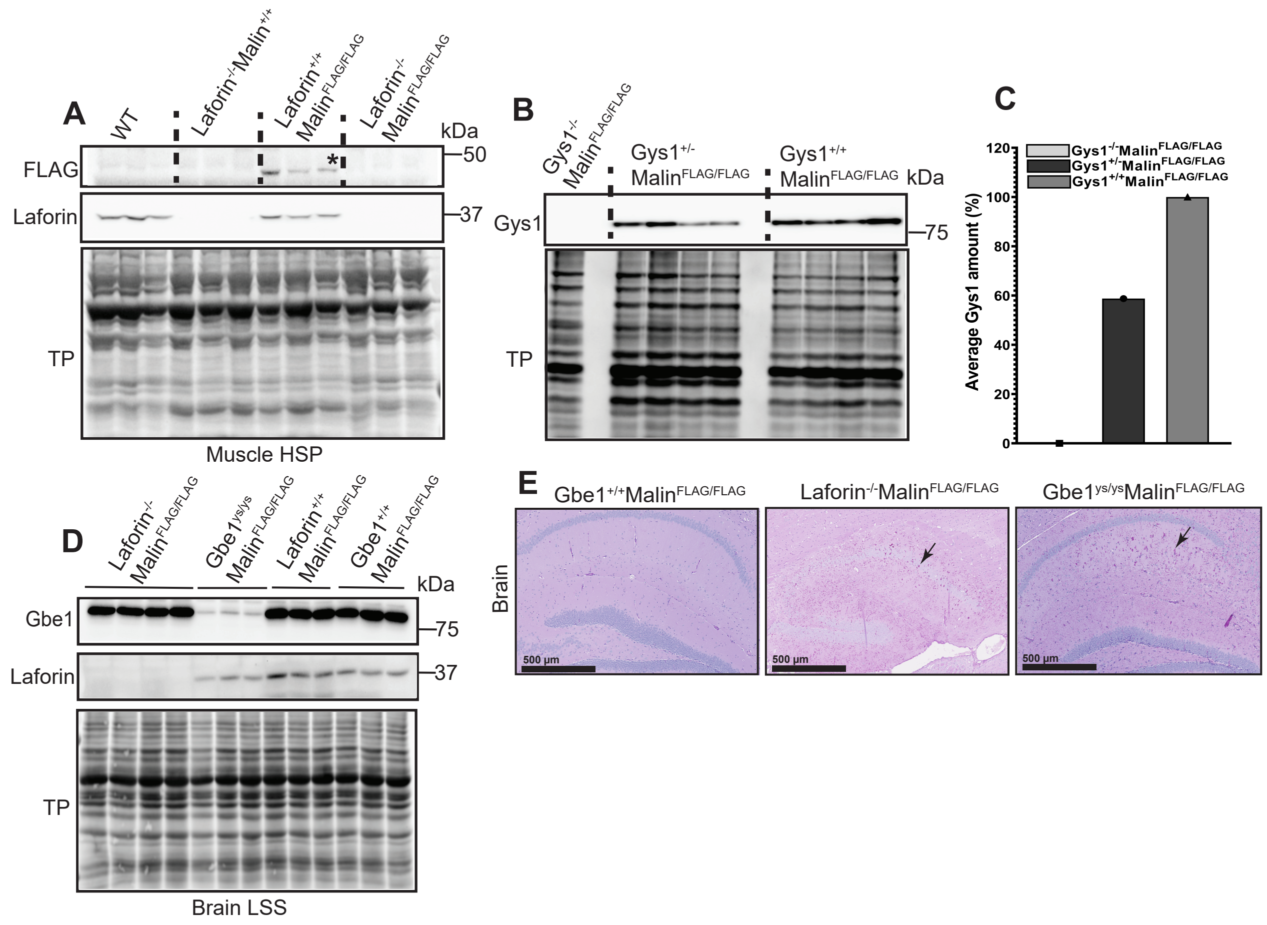

Fig. S3. Laforin mediated glycogen association of malin validated using several mouse models. (A) Supplementary to figure 4.A, immunoblot analysis with HSP fractions from muscle tissues of indicated genotypes of female mice shows FLAG-malin bands only in Laforin$^{+/+}$Malin$^{FLAG/FLAG}$ mice but not in Laforin$^{-/-}$Malin$^{FLAG/FLAG}$, Laforin$^{-/-}$Malin$^{+/+}$ or Laforin$^{+/+}$Malin$^{+/+}$ (WT) mice. (B) Immunoblot analysis with LSS fractions from muscle tissues of the indicated genotypes of male mice shows absence or reduced Gys1 band intensity in Gys1$^{-/-}$Malin$^{FLAG/FLAG}$ and Gys1$^{+/-}$Malin$^{FLAG/FLAG}$ mice respectively compared with Gys1$^{+/+}$Malin$^{FLAG/FLAG}$ mice. (C) Quantification of Gys1 protein band from (B). Average protein amount is calculated from n=4 with Gys1$^{+/+}$Malin$^{FLAG/FLAG}$ and Gys1$^{+/-}$Malin$^{FLAG/FLAG}$ genotypes. For Gys1$^{-/-}$Malin$^{FLAG/FLAG}$, only one mouse was used due to difficulties in obtaining such genotype (Pederson et al., 2004). As expected, partial

Disease Models & Mechanisms • Supplementary information

knockout of *Gys1* gene in FLAG-malin mouse ($Gys1^{+/-}Malin^{FLAG/FLAG}$) resulted in ~40% reduction in protein amount compared to $Gys1^{+/+}Malin^{FLAG/FLAG}$, whereas complete knockout of the gene ($Gys1^{-/-}Malin^{FLAG/FLAG}$) resulted in no detectable Gys1 protein. (D) Immunoblot analysis using brain LSS fractions from indicated genotypes of male mice. As expected, the amount of Gbe1 protein is reduced in $Gbe1^{ys/ys}Malin^{FLAG/FLAG}$ brains but not in $Laforin^{-/-}Malin^{FLAG/FLAG}$, $Laforin^{+/+}Malin^{FLAG/FLAG}$ or $Gbe1^{+/+}Malin^{FLAG/FLAG}$ ones (upper blot). Middle blot shows that laforin is not detected in $Laforin^{-/-}Malin^{FLAG/FLAG}$ mice, but is in all other indicated genotypes. (E) Representative images from PAS-D stained brains from 7 month-old $Gbe1^{ys/ys}Malin^{FLAG/FLAG}$ and 12 month-old $Laforin^{-/-}Malin^{FLAG/FLAG}$, $Laforin^{+/+}Malin^{FLAG/FLAG}$ are shown. The images represent n=2 per group where male mice were used. Arrows indicate polyglucosan bodies. TP, total protein.

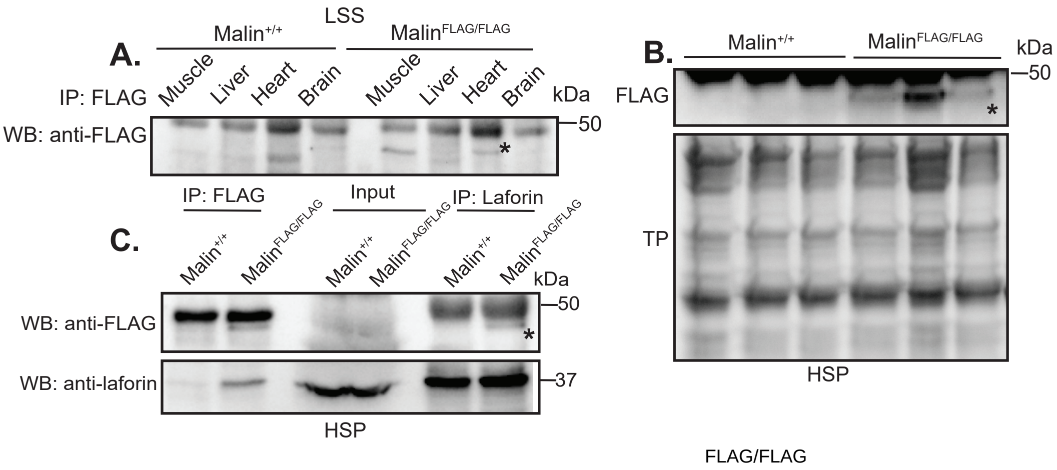

Fig. S4. (A) Immunoprecipitation of FLAG-malin in multiple tissues of female mice. Lysates from the indicated tissues of female Malin$^{FLAG/FLAG}$ and Malin$^{+/+}$ mice were immunoprecipitated using anti-FLAG antibody and immunoblot analysis was carried out using anti-FLAG antibody. Representative image from two independent experiments is shown. (B) Immunoblot analysis using anti-FLAG with HSP fraction from muscle tissues from the indicated genotypes of female mice (n=3) shows FLAG-malin band in Malin$^{FLAG/FLAG}$ mice but not in Malin$^{+/+}$ mice (upper blot). Lower blot shows total protein (TP). (C) Co-immunoprecipitation of FLAG-malin and laforin at the HSP fraction. HSP fractions from muscle tissues of female Malin$^{FLAG/FLAG}$ and Malin$^{+/+}$ mice were immunoprecipitated using anti-FLAG or anti-laforin antibodies and the HSP fraction (input) or the immunoprecipitates were immunoblotted with anti-FLAG (upper blot) and anti-laforin antibodies (lower blot). In all blots, FLAG-malin band is indicated by an asterisk.

Disease Models & Mechanisms • Supplementary information

Table S1. Genotyping primers used in this study to validate mouse models

Primer name	Purpose	Sequence (5'-3′)	Amplicon size (bp)
FL-malin-F<br>FL-malin-R	Detect *Nhlrc1* with or without FLAG insertion	ACTGCGTCGTGCGTCCGCC<br>GCTGCCACCGCCGTCGCCTC	153 ($Nhlrc1^{+}$), 177 ($Nhlrc1^{fl}$)
CAG-Cre-F<br>CAG-Cre-R	Detection of *CAG-Cre* transgene	AGGTTCGTTCACTCATGGA<br>TCGACCAGTTTAGTTACCC	235 (Cre^{+})
Exon remove-F<br>Exon remove-R	Detection of exon 6-8 excision of *Gys1*	AGGGTCAAGTAGCGGTGTTG<br>AACGCCCTACCATGAGCTAC	2.1 Kbp ($Gys1^{+}$) and 293 ($Gys1^{-}$)

Disease Models & Mechanisms • Supplementary information

Table S2. Primers used in qPCR study for gene expression

Gene	Primers	Sequence (5′-3′)
Epm2a	Epm2a_F	CTGGGAAGGAAATGGACCTCA
	Epm2a_R	GGCCTCAATCCAGTGTCCTA
Nhlrc1	Nhlrc1_F	CTGCTAGCGTCATGGGCATA
	Nhlrc1_R	CACAAAGTGCTTCAGGCGTC
Gbe1	Gbe1_F	GATTCTGACGCAGCGGAGTA
	Gbe1_R	GAAGGATGAGAGCCACTCGG
Pygm	Pygm_F	CTCCAGGACATCATCCGACG
	Pygm_R	GCTGGATGGCCACCTTATCA
Pygb	Pygb_F	CATGATCCAGTTGTGGGCGA
	Pygb_R	AGCCGCTGGGATCACTTTC
Rbck1	Rbck1_F	GCAGGGAGTACCAAGACGAC
	Rbck1_R	CAGCAGATCTCAGTGTGGCA
Gys1	Gys1_F	GTCCTCGCTTCCAGGATTGG
	Gys1_R	TGTAGATGCCACCCACCTTGT
Ppp1r3c	Ppp1r3c_F	ATGGAAACCTGACGGAGTGC
	Ppp1r3c_R	CAAGTTCTCCACTCTCCCCC
Agl	Agl_F	CGACCGAAACATGAAGGACG
	Agl_R	CAGCAGACCCATCTCTTGGAG
Sharpin	Sharpin_F	CCTGGGGCGGTCAGTTT
	Sharpin_R	GGAAGTGCACGCTGAAGGTT
HOIP	HOIP_F	AGAGAGCCTAGACCCCGATG
	HOIP_R	AAGCCAAAGGAACACTGGGC
Actin	Actin_F	AGTGTGACGTTGACATCCGTA
	Actin_R	GCCAGAGCAGTAATCTCCTTC
Rpl4	Rpl4_F	CCCTTACGCCAAGACTATGC
	Rpl4_R	TGGAACAACCTTCTCGGATT
Oazl	Oazl_F	AGGGCAGTAAGGACAGTTTTG
	Oazl_R	TCTCACAATCTCAAAGCCAAG

Disease Models & Mechanisms • Supplementary information
